# Molecular Cloning of the *scd1* Gene and Its Expression in Response to Feeding Artificial Diets to Mandarin Fish (*Siniperca chuatsi*)

**DOI:** 10.3390/genes15091211

**Published:** 2024-09-16

**Authors:** Jiangjiang Wang, Lihan Zhang, Xiaowei Gao, Yanfeng Sun, Chunlong Zhao, Xiaotian Gao, Chengbin Wu

**Affiliations:** 1 Ocean College, Hebei Agricultural University, Qinhuangdao 066003, China; marimo0727@163.com (J.W.); hyxyzhlh@hebau.edu.cn (L.Z.); hygxw123@163.com (X.G.); sunyanfeng@hebau.edu.cn (Y.S.); 2 Hebei Key Laboratory of Aquaculture Nutritional Regulation and Disease Control, Qinhuangdao 066003, China; 3 Hebei Academy of Ocean and Fishery Sciences, Qinhuangdao 066200, China; zhaochunlong1968@163.com

**Keywords:** *scd1*, molecular cloning, tissue expression, *Siniperca chuatsi*

## Abstract

**Background/Objectives**: Stearoyl-coenzyme A desaturase 1 (SCD1) plays a crucial role in fatty acid metabolism. However, its roles in the feeding habit transformation of mandarin fish (*Siniperca chuatsi*) remain largely unknown. **Methods:** Juvenile mandarin fish (10.37 ± 0.54)g were trained to feed on an artificial diet and then divided into artificial diet feeders and nonfeeders according to their feed preference. Afterwards, the *scd1* gene of mandarin fish (*Sc-scd1*) was identified and characterized, and its transcription difference was determined between *S. chuatsi* fed live artificial diets and those fed prey fish. **Results:** Our results show that *Sc-scd1* coding sequence is 1002 bp long, encoding 333 amino acids. The assumed *Sc*-SCD1 protein lacks a signal peptide, and it contains 1 N-linked glycosylation site, 24 phosphorylation sites, 4 transmembrane structures, and 3 conserved histidine elements. We found that *Sc*-SCD1 exhibits a high similarity with its counterparts in other fish by multiple alignments and phylogenetic analysis. The expression level of *Sc-scd1* was detected with different expression levels in all tested tissues between male and female individuals fed either live prey fish or artificial diets. **Conclusions:** In particular, the *Sc-scd1* expression level was the highest in the liver of both male and female mandarin fish fed artificial diets, indicating that *scd1* genes may be associated with feed adaption of mandarin fish. Taken together, our findings offer novel perspectives on the potential roles of *scd1* in specific domestication, and they provide valuable genetic information on feeding habits for the domestication of mandarin fish.

## 1. Introduction

Mandarin fish (*Siniperca chuatsi*), widely distributed in China, Vietnam, and Korea, is one of the main economic freshwater species due to its fast growth, tasty flesh, and rich nutrients [1]. Recently, the aquaculture scale of mandarin fish in China has been developing rapidly, reaching 477,592 tons in 2023, with a 18.95% increase from 2022 [2]. It is well known that *S. chuatsi* has a peculiar habit of feeding on live prey fish throughout its life. However, in recent decades, *S. chuatsi* has been proven to possess a considerable amount of adaptability to artificial diets using specific domestication and culture techniques [3,4]. Studies on feeding artificial diets to mandarin fish have mainly focused on growth performance, muscle nutrient composition, and feed utilization; thus, studies on the metabolic response of *S. chuatsi* to artificial diets remain scarce [5,6,7]. Growing evidence has demonstrated that, in *S. chuatsi*, artificial diets increase glycogen and lipid accumulation, which induce oxidative stress and inflammation, thus threatening its health [8,9]. Notably, an appropriate dietary carbohydrate-to-lipid ratio has been found to effectively improve feed intake, upregulate fish appetite, and reduce lipid accumulation in the liver of *S. chuatsi* [10]. Economically, the domestication of *S. chuatsi* to feed on an artificial diet is beneficial for promoting its intensive breeding, improving economic benefits, and minimizing ecological damage. Therefore, the metabolic difference in mandarin fish fed an artificial diet needs much more attention.

Stearoyl-coenzyme A desaturases (SCDs) play a pivotal role in the de novo synthesis of monounsaturated fatty acids (MUFAs), contributing to the formation of intricate lipid structures encompassing acyl lipids, membrane-spanning phospholipids, cholesterol esters, as well as triglycerides [11]. A previous study found that a high-carbohydrate diet could induce the expression of the *scd1* gene, resulting in increased triglyceride accumulation in the liver and adipose tissue [12]. Likewise, in teleosts, the dietary nutritional status was also found to influence SCD1 expression [13,14,15,16]. Ntambi et al. (2002) found that mice genetically lack SCD1 exhibited resistance to adiposity and hepatic steatosis induced by a high-carbohydrate diet, suggesting its function in maintaining lipid homeostasis [17]. He et al. (2021) suggested that lipid metabolism contributed to domestication using an artificial diet, as they found that the *Scd* gene was significantly induced in mandarins fed an artificial diet [18]. Apart from the abovementioned functions of SCD1, studies have also shown that SCD1 plays an important role in diverse cellular functions [19,20]. Although many studies have proven that SCD1 is critical in various physiological and biochemical processes, the functions of SCD1 in mandarin fish, especially their potential to regulate feed preference, are not fully understood.

SCD1, a key enzyme in fatty acid metabolism, plays crucial roles in cellular metabolism, stress, and immune regulation in mammals, but information on its roles in teleosts is limited [21]. A recent study in our lab indicated that *S. chuatsi* fed live prey fish and those fed an artificial diet presented differences in *scd1* expression, according to a WGCNA analysis [22]. Moreover, we identified the existence of an amino acid mutation of the *scd1* gene in a whole-gene comparison between mandarin fish fed live prey fish (lipid content of 8.46% and protein content of 19.22% on a dry matter basis) and those fed an artificial diet (lipid content of 10.5% and protein content of 48.6% on a dry matter basis), which revealed that SCD1 may play crucial roles in the adaptation of *S. chuatsi* to feed conversion. Thus, we cloned and characterized the *scd1* gene in *S. chuatsi*, identified its expression pattern in selected tissues, and further analyzed the differences in its expression at the transcription level response to different feeding diets. Valuable genetic information in terms of feeding habits is provided for further studies on the domestication of *S. chuatsi*.

## 2. Materials and Methods

### 2.1. Animal Ethics

This study was conducted following the Laboratory Animal Welfare Guidelines of China (GB/T 35892-2018) [23], and the experiment was approved by the Animal Experimentation Ethics Committee of Hebei Agricultural University (Grant No. 2023075).

### 2.2. Fish Sampling

Juvenile mandarin fish (10.37 ± 0.54) g were obtained from Chizhou Yijue Feed Co., Ltd. Anhui, China, and they were temporarily placed in a rectangle raft net cage for two weeks of acclimation. Training for the feeding habit transformation of the mandarin fish was performed as described by Liang et al. [24]. Briefly, the mandarin fish were fed the fry of live India mrigal (*Cirrhinus mrigala*) as prey fish and the fry of frozen India mrigal (lipid content of 8.46% and protein content of 19.22% on a dry matter basis) as dead prey fish. During training, the fish were visually categorized into feeders and non-feeders based on plumpness and emaciation; then, the categorized mandarin fish were respectively fed live prey fish and artificial diets (lipid content of 10.5% and protein content of 48.6% on a dry matter basis) to satiation two times daily for 30 days.

**Figure 1:**
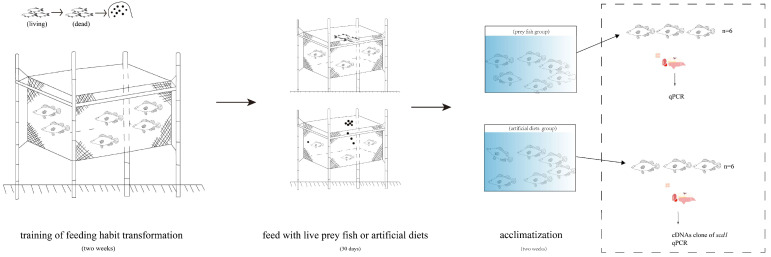
Graphical overview of the experimental procedure.

After the 2-week acclimatization, 12 fish (6 female and 6 male fish) in each treatment (live prey fish treatment and artificial diet treatment) were randomly suppressed using MS-222 (100 mg L^−1^; cat: D0063637, Shanghai Amperexperiment Technology Co., Anpel, Shanghai, China). Ten tissues, namely, the heart, brain, liver, muscle, gill, intestines, kidneys, stomach, gonads, and spleen, were separated on ice, then rapidly frozen for RNA isolation. The experimental procedure is shown in Figure 1.

### 2.3. Total RNA Isolation and cDNA Synthesis

The total RNA was obtained using TRIzol RNA reagent (cat: 15596026, Invitrogen, Carlsbad, CA, USA) as described by Zhang et al. [25]. mRNA reverse transcriptions were conducted with a Hifair^®^ III 1st Strand cDNA Synthesis Super Mix (cat: 11141ES10, YEASEN, Shanghai, China).

### 2.4. cDNAs Cloning of scd1 Gene

The partial gene sequence of *scd1* was obtained from the genome database of mandarin fish, which was reported in a previous work [26], and from transcriptome data from our lab. The *scd1* gene sequence of zebrafish (Genebank No. NM_198815) was chosen as a reference. The core fragments of mandarin fish *scd1* (*Sc*-*scd1*) were amplified using PCR (Table 1). The PCR program was designed by Zhang et al. [25]. We used an EZNA gel extraction kit (cat: D2500-01, Omega Bio-Tek, Norcross, Georgia, USA) to purify target fragments.

### 2.5. Sequence Analyses and Data Processing

Physicochemical properties and features of SCD1 in mandarin fish were predicted by using online software (Table 2). And the SCD amino acid sequences of other fish were obtained from the NCBI. The percentages of SCD protein similarity were analyzed using Mega 11.0 and DNAMAN software. The program Align of the DNAMAN package with the ClustalW method was used to align multiple protein sequence of SCDs. MEGA 6.0 with the neighbor-joining (NJ) method based on the JTT + G model [27] was performed to construct a phylogenetic tree, in which the confidence was determined with 1000 bootstrap replicates. Finally, the structures of *scd* genes in different vertebrates were examined using a comparative genomic survey, and the genome databases of other vertebrate species, including *Homo sapiens* (NM_001037582.3, NCBI), *Mus musculus* (NM_009128.2, NCBI), *Gallus gallus* (NM_204890.2, NCBI), and *Alligator sinensis* (XM_006014792.3, NCBI), were used as references.

### 2.6. Tissue Distribution Levels of Sc-scd1

The mRNA expression levels of *Sc-scd1* in the tissues of each treatment were determined using qPCR. An equal amount of RNA of 6 fish of the same gender in each treatment was pooled and used to construct libraries for the qPCR. The qPCR was performed as previously method [28]. Primers for specific gene were synthesized by Sangon Biotechnology Co. (Beijing, China) (Table 1). The comparative ΔΔCt method was used for analyzing the relative expression level, and *β-actin* was selected to normalize. Each experiment was replicated three times.

### 2.7. Statistical Analysis

Statistical analyses were conducted using SPSS 26.0 software (SPSS, Michigan Avenue, Chicago, IL, USA). Data are shown as means ± standard error of mean (SEM). Data were examined using a one-way ANOVA and measured using Duncan’s multiple range tests. Statistically significant was *p* < 0.05. Graphs were obtained using Origin 2021 (OriginLab Inc., Northampton, MA, USA).

## 3. Results

### 3.1. Comparative Syntenic and Structural Analyses of scd1 Genes in Vertebrates

**Figure 2:**
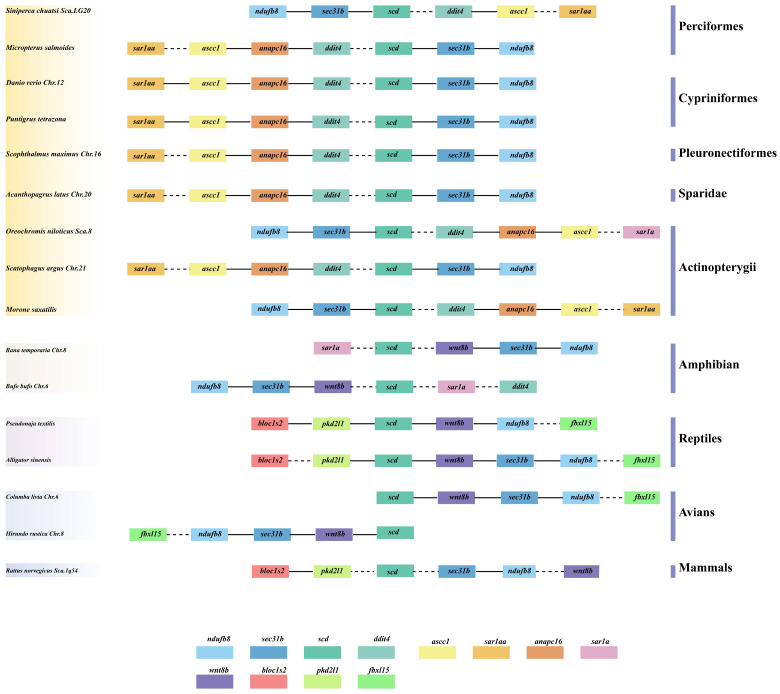
*scd* gene synteny comparisons in different genomes of vertebrate. The colorful blocks, intergenic regions; the solid and dotted lines, without genes.

The analyses of comparative syntenic and structural genomics in *scd1* genes were performed among different vertebrates, including avians (barn swallow and rock pigeon), mammals (Norway rat), amphibians (common frog and common toad), reptiles (Chinese alligator and eastern brown snake), cypriniformes (zebrafish and sumatra barb), perciformes (mandarin fish and largemouth bass), pleuronectiformes (turbot), Sparidae (yellowfin seabream), and Actinopterygii (nile tilapia, *Scatophagus argus*, and striped sea-bass). Similar to the tetrapod lineage, these results show that a single copy of the *scd1* gene was extensively transcribed in teleost species (Figure 2), and the *scd1* gene occurred in all examined genomes of different fish. The gene cluster *scd-sec31b-ndufb8* was found to be highly conserved in mammals and some teleost species, and the gene cluster *blocls2-pkd2l1-scd* was found to be highly conserved between mammals and reptiles (Figure 2). In addition, the gene cluster *scd-*(*x*)*-sec31b-ndufb8* was found to be conserved in vertebrates. Interestingly, a different gene, *anapc16*, was detected in all teleosts selected, except for mandarin fish, in which the *anapc16* gene was located between *ascc1* and *ddit4* (Figure 2).

**Figure 3:**
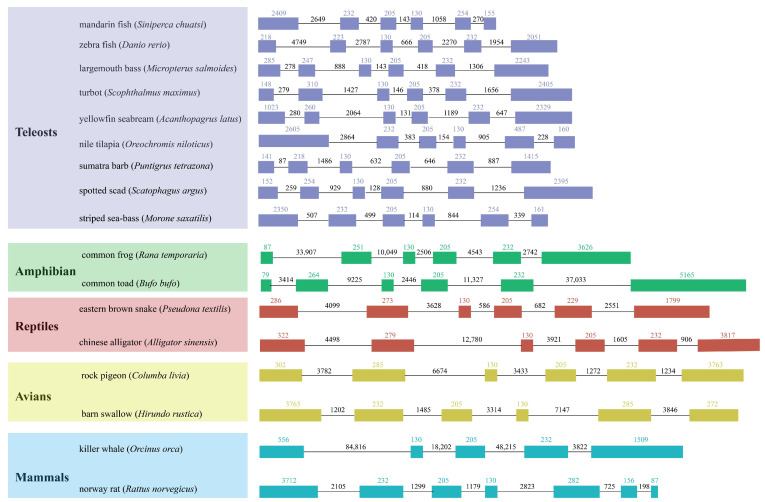
*scd* gene structural comparisons in different vertebrates. Blocks, exons; solid lines, introns; numbers above the colorful boxes, the length of the coding sequences; lines, length of the introns.

An examination of the gene structure of vertebrate *scd1* revealed that the numbers of *scd1* gene exon and intron varied among vertebrates (Figure 3). The gene structure of *Scd5* in killer whale (*Orcinus orca*) contained five exons and four introns, while the *scd1* gene in *Rattus norvegicus* possessed seven exons and six introns (Figure 3). *Scd1* was widespread in teleosts and showed a high degree of conservation, with six exons and five introns. In addition, three exons of the same length as in teleosts were present in *scd1* in Norway rat and in the same positions as those in mandarin fish, Nile tilapia, and striped sea-bass, being located in the second to fourth positions (Figure 3). Moreover, three exons with a similar length were observed in teleosts, but the length of the introns varied in different species (Figure 3).

### 3.2. Tissue Distribution Pattern of Sc-scd1

**Figure 4:**
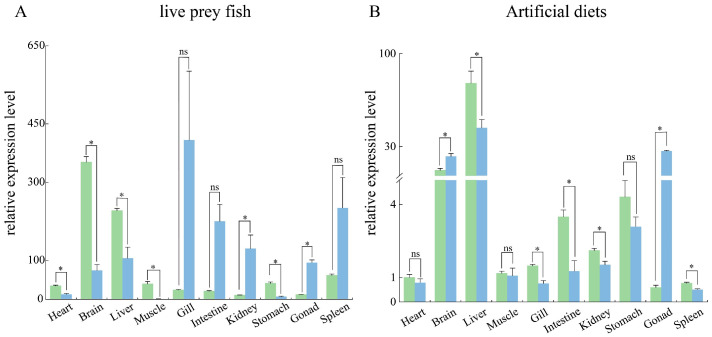
*scd1* gene tissue distribution levels of *S. chuatis*. (**A**), Live prey fish group. (**B**), Artificial diet group. In (**A**,**B**), green columns represent females, and blue columns represent males. *: significant differential expression of *scd1* between male and female tissues.

Ten selected tissues, namely the heart, brain, liver, muscle, gills, intestine, kidney, stomach, gonads, and spleen, were examined. The results show that the *Sc-scd1* gene was extensively expressed in different tissues, with variation among the different sexes and tissues. The expression levels of *scd1* in the *S. chuatis* fed live prey fish varied between the females and males. In females, the highest expression level of *Sc-scd1* was in the brain, followed by the liver (Figure 4A). However, in males, the highest was in the gill, followed by the intestines and spleen, while the lowest was found in muscle (Figure 4A). The *scd1* mRNA expression levels in the mandarin fish fed commercial diets differed between the male and female tissues (Figure 4A). The *Sc-scd1* mRNA expression levels were significantly increased in the heart, brain, liver, muscle, and stomach but significantly decreased in the kidney and gonad in females (Figure 4A). The highest expression level of *Sc-scd1* was in the liver of females, followed by the brain (Figure 4B). In contrast, the highest was in the liver of males, followed by the testis and brain, while the lowest was found in spleen (Figure 4B). As shown in Figure 4B, the *scd1* mRNA expression levels in the mandarin fish fed artificial diets differed between the male and female tissues. The *Sc-scd1* mRNA expression levels in the females were significantly increased in the liver, gill, intestine, kidney, and spleen but significantly decreased in the brain and gonad (Figure 4B).

### 3.3. Molecular Cloning and Characterization of Sc-scd1

**Figure 5:**
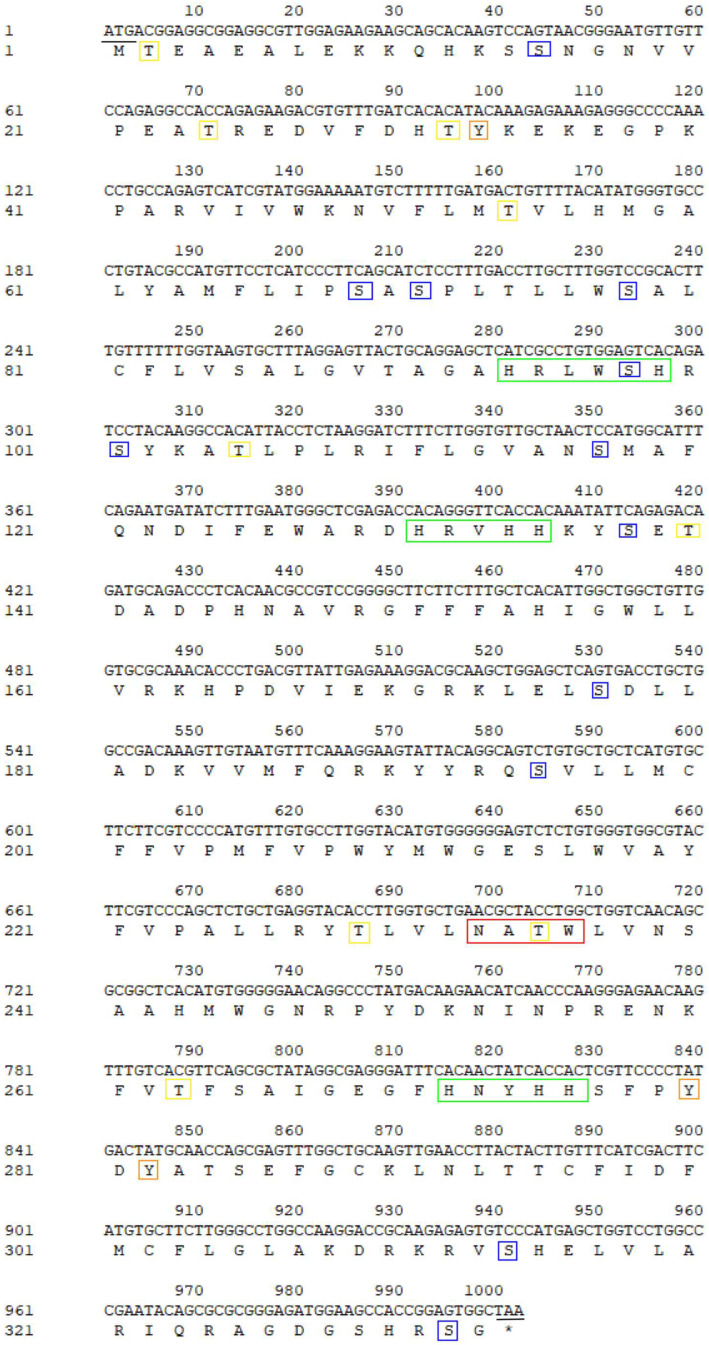
Nucleotide sequence encoding SCD1 in *S. chuatsi* and the deduced amino acid sequence. Positions of the nucleotide and amino acid (left number), initiation codon and termination codon (underline), stop codon (black asterisk, *), three near-consensus histidine motifs (green solid box), putative serine phosphorylation sites (blue solid boxes), tyrosine phosphorylation sites (orange solid boxes), threonine phosphorylation sites (yellow solid boxes).

**Figure 6:**
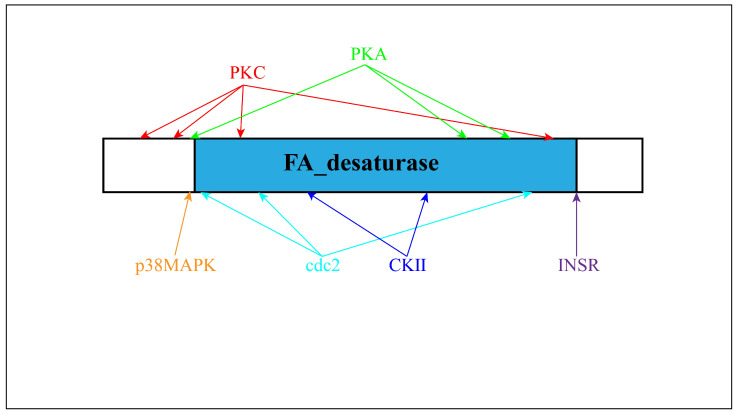
Schematic diagram of domains of *S. chuatsi scd1* gene. A fatty acid desaturase is shown in blue. Four conserved PKC phosphorylation sites (red), three conserved PKA phosphorylation sites (green), three conserved cdc2 phosphorylation sites (cyan), two conserved CKⅡ phosphorylation sites (blue), binding sites for p38MAPK (orange), and INSR are indicated (purple).

**Figure 7:**
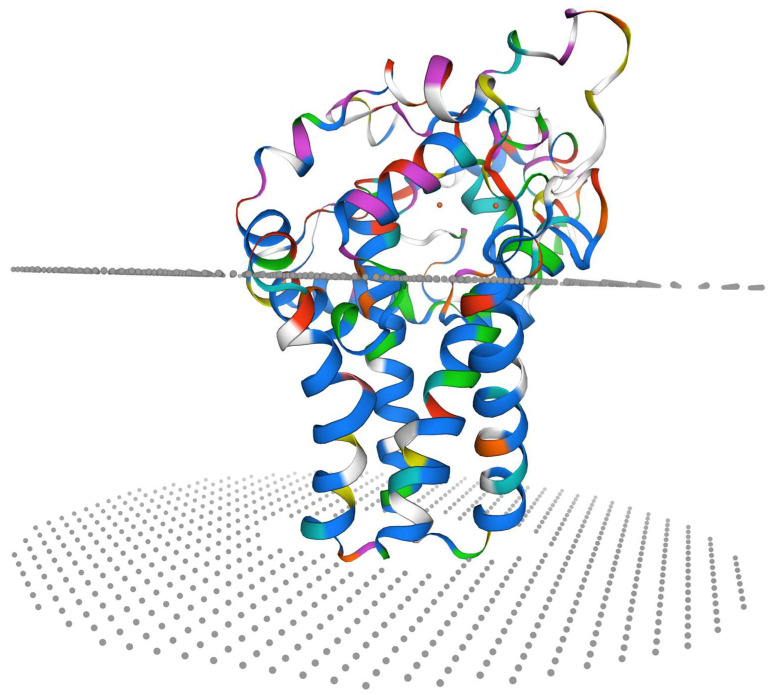
Tertiary structure prediction of SCD1 protein in *S. chuatsi*.

The ORF of *Sc-scd1* was 1002 bp in length, and it was predicted to encode 333 amino acid residues (Figure 5). We calculated that its putative molecular weight was 38.43 kDa, and its theoretical isoelectric point was 9.25. Table 3 shows the amino acid compositions. Four transmembrane domains were identified in the *Sc*-SCD1 protein with no signaling peptide. Meanwhile, one N-linked glycosylation site (NATW, aa: 233–236) was found. Twelve conserved phosphorylation sites were identified at serine (aa: 15, 69, 71, 78, 98, 101, 117, 138, 177, 195, 314, and 329) with NetPhos-3.0. Additionally, three phosphorylation sites at tyrosine (aa: 33, 280, and 282) and nine phosphorylation sites at threonine (aa: 2, 24, 32, 54, 105, 140, 229, 235, and 263) were identified using NetPhos-3.0. In addition, the conserved kinase binding sites were predicted to be accessible with NetPhos-3.0 (Figure 5). Positions 75 to 280 of the amino acid sequence of *Sc*-Scd1 aligned with the conserved structural domain of fatty acid desaturase (sequence number: pfam00487), which belongs to the FA_desaturase superfamily (Figure 6). A PSORT subcellular localization analysis showed that *Sc*-Scd1 was most likely to be distributed in the cytoplasm (Table 4). The prediction of the secondary structure of *Sc*-Scd1 using SOPMA showed that the protein consisted of α-helices (126 aa), extended strands (35 aa), and free irregular coils (172 aa), which accounted for 37.84%, 10.51%, and 51.65%, respectively. Swiss-Model predictions showed that *Sc*-SCD1 is a monomeric protein, with its tertiary structure mainly consisting of irregular curls, which was consistent with the secondary structure prediction (Figure 7).

### 3.4. Alignment of SCD Protein Sequence

**Figure 8:**
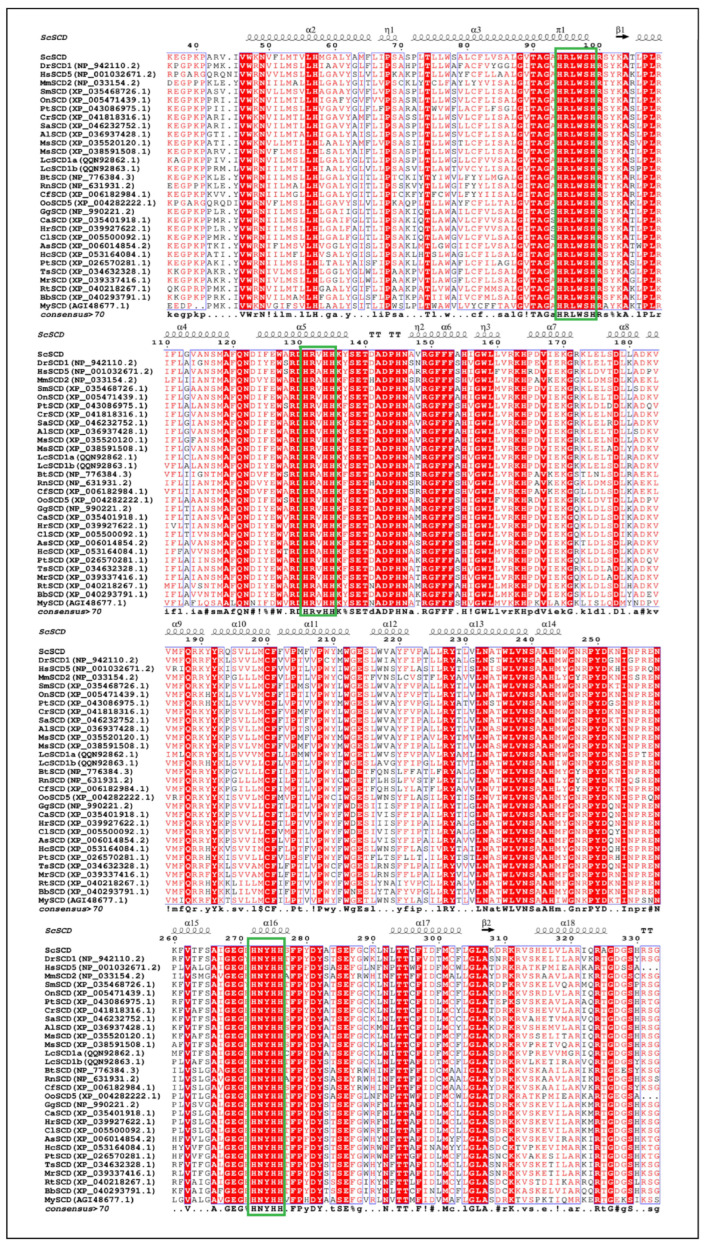
Alignment of SCD1 amino acid sequence of *S. chuatsi* with that of different species. Solid green boxes represent conserved histidine components. Abbreviated species names and full names are as follows: ScSCD: *S. chuatsi*; DrSCD1 (NP_942110.2): Danio rerio; HsSCD5 (NP_001032671.2): Homo sapiens; MmSCD2 (NP_033154.2): Mus musculus; SmSCD (XP_035468726.1): Scophthalmus maximus; OnSCD (XP_005471439.1): Oreochromis niloticus; PtSCD (XP_043080975.1): Puntigrus tetrazona; CrSCD (XP_041818316.1): Chelmon rostratus; SaSCD (XP_046232752.1): Scatophagus argus; AlSCD (XP_036937428.1): Acanthopagrus latus; MsSCD (XP 035520120.1): Morone saxatilis; MsSCD (XP 038591508.1): Micropterus salmoides; Lc-SCD1a (QQN92862.1): Larimichthys crocea; Lc-SCD1b (QQN92863.1): Larimichthys crocea; BtSCD (NP_776384.3): Bos taurus; RnSCD (NP_631931.2): Rattus norvegicus; CfSCD (XP_006182984.1): Camelus ferus; OoSCD5 (XP_004282222.1): Orcinus orca; GgSCD (NP_990221.2): Gallus gallus; CaSCD (XP_035401918.1): Cygnus atratus; HrSCD (XP_039927622.1): Hirundo rustica; ClSCD (XP_005500092.1): Columba livia; AsSCD (XP_006014854.2): Alligator sinensis; HcSCD (XP_053164084.1): Hemicordylus capensis; PtSCD (XP_026570281.1): Pseudonaja textilis; TsSCD (XP_034632328.1): Trachemys scripta elegans; MrSCD (XP_039337416.1): Mauremys reevesii; RtSCD (XP_040218267.1): Rana temporaria; BbSCD (XP_040293791.1): Bufo bufo; MySCD (AGI48677.1): Mizuhopecten yessoensis.

The SCD protein was highly conserved in vertebrates, with similar consensus motifs, involved in three histidine motifs (HRLWSH, HRV/AHH, and HNYHH). Additionally, we predicted that the putative *Sc*-SCD1 contained four transmembrane domains (aa: 44-66, 76–98, 195–212, and 216–238), three histidine motifs (aa: 94–100, 131–135, and 272–276) (Figure 8), and two ion binding sites (Figure 7).

A similarity analysis indicated that *Sc*-SCD1 shared a 61.20–73.9% identity with its homologs in human (70.61%), Norway rat (65.08%), cattle (61.71%), killer whale (72.05%), chicken (73.38%), barn swallow (73.38%), Chinese alligator (70.61%), eastern brown snake (70.45%), Reeves’s turtle (73,70%), and frog (73.38%). On the contrary, *Sc*-SCD1 shared a higher similarity with its homolog in teleosts, including zebrafish (76.61%), turbot (83.98%), largemouth bass (88.51%), yellowfin seabream (91.30%), and Nile tilapia (93.12%) (Figure 8).

### 3.5. SCD Phylogenetic Analysis

**Figure 9:**
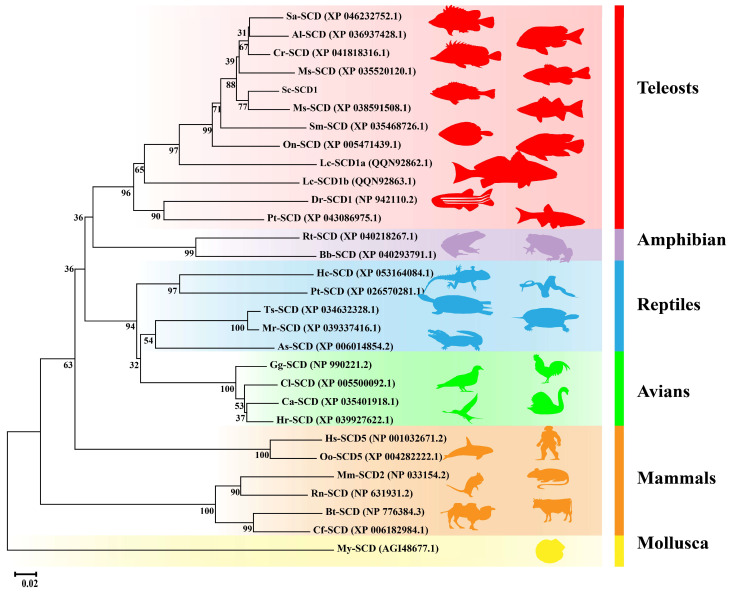
Phylogenetic tree based on the amino acid sequence of SCD1 in mandarin fish and in other vertebrate species. The cluster of Yesso scallop (*Mizuhopecten yessoensis*) SCD1 was used as the outgroup. Abbreviated species names and full names are as follows: Sa-SCD: Scatophagus argus; Al-SCD: Acanthopagrus latus; Cr-SCD: Chelmon rostratus; Ms-SCD (XP 035520120.1): Morone saxatilis; Sc-SCD: Siniperca chuatsi; Ms-SCD (XP 038591508.1): Micropterus salmoides; Sm-SCD: Scophthalmus maximus; On-SCD: Oreochromis niloticus; Lc-SCD1a: Larimichthys crocea; Lc-SCD1b: Larimichthys crocea; Dr-SCD1: Danio rerio; Pt-SCD: Puntigrus tetrazona; Rt-SCD: Rana temporaria; Bb-SCD: Bufo bufo; Hc-SCD: Hemicordylus capensis; Pt-SCD: Pseudonaja textilis; Ts-SCD: Trachemys scripta elegans; Mr-SCD: Mauremys reevesii; As-SCD: Alligator sinensis; Gg-SCD: Gallus gallus; Cl-SCD: Columba livia; My-SCD: Mizuhopecten yessoensis; Hr-SCD: Hirundo rustica; Hs-SCD5: Homo sapiens; Oo-SCD5: Orcinus orca; Mm-SCD2: Mus musculus; Rn-SCD: Rattus norvegicus; Bt-SCD: Bos taurus; Cf-SCD: Camelus ferus; Ca-SCD: Cygnus atratus.

The results of the NJ phylogenetic tree show that SCD1 was divided into five major groups of amphibians, reptiles, avians, mammals, and teleosts (Figure 9). SCD1 was further clustered into two adjacent clades in the group of teleosts. The SCD1 in Cypriniformes (zebrafish and Sumatra barb) was clustered into a single unit, which is evolutionarily distantly related to fishes of other families. Meanwhile, *Sc*-SCD1 shared a proximate Association with its counterpart in largemouth bass (*Micropterus salmoides*) (Figure 9).

## 4. Discussion

In the present study, *Sc-scd1* showed a highly conserved synteny and gene structure with those in other vertebrates. The genes *sec31b* and *ddit4*, which were located on chromosome 20, were confirmed as the flanking genes of *Sc-scd1*, differing from those in mammals (Norway rat), avians (barn swallow and rock pigeon), amphibians (frog and toad), and reptiles (Chinese alligator and eastern brown snake). This difference may be due to gene rearrangement and/or gene deletion during the process of evolution [29]. Moreover, *Sc-scd1* exhibited a conserved gene structure, characterized by the maintenance of the numbers of exons and introns, while the intron lengths varied, which suggested that *scd1* may have undergone a distinct evolutionary trajectory, independent from other species. Taken together, the conservation of the gene arrangement and exon sizes suggests that the *scd1* gene may have similar functions among teleost species, while the independent evolutionary history suggests that functional differentiation may also exist among teleost species.

The liver, as an important site for lipid synthesis in fish, is involved in the regulation of lipid metabolism in vivo. In the present study, *Sc-scd1* was expressed in all tissues of both female and male *S. chuatis* fed an artificial diet, with the highest expression level being in the liver. The high expression level of *Sc-scd1* in the liver may be related to its involvement in the regulation of fatty acid synthesis and metabolism in the liver [30]. As reported in salmonid trout, intestinal epithelial cells are involved in the endogenous pathway of lipid transport [31]. It has been shown that celiac granules in the intestine of Atlantic cod (*Gadus morhua*) are the main transporters of intestinal lipids [32]. The high expression level of *scd1* in the intestine of male *S. chuatsi* fed prey fish may imply the presence of fat transporter carriers in the intestine of *S. chuatsi* similar to those in Atlantic cod. Additionally, our results show that the *Sc-scd1* gene was extensively transcribed in different tissues and varied among sexes, similar to other studies in fish, including black seabream and gefilte tilapia [33,34]. However, in tilapia, *scd1* has only been found to be expressed in the liver [35]. The distribution pattern of *scd1* in different tissues of *S. chuatsi* being different from that of other fishes may be the result of species-specific regulation, related to the different growth environments of the species and the degree of dependence on *scd1*. Previous studies have shown that the domestication of mandarin fish via artificial feeding adaptively changes not only the histological structure but also the expression levels of numerous genes, including digestive enzyme- and lipid synthesis-related genes [4,9,36]. *Scd1*, one of the key genes for lipid metabolism, may play an important role in feed domestication. Studies have shown that dietary nutrient levels affect *scd1* expression in both mammals and fishes [14,16,37]. In the present study, feeding with artificial diets significantly increased the gene expression level of *Sc-scd1* in the liver, stomach, gonad, and brain in both the female and male mandarin fish, suggesting that the feed domestication of *S. chuatsi* contributed to the better capacity for glycerolipid metabolism and unsaturated fatty acid biosynthesis. Taken together, these results demonstrate that *Sc-scd1* expression levels are altered by different dietary nutrient levels and sexes, indicating the crucial roles of *scd1* in the feeding habit transformation of mandarin fish.

In mammals, it has been reported that SCD includes SCD1, SCD2, SCD3, SCD4, and SCD5, of which SCD1 is the predominant isoform [38]. However, only one variant of the *Scd* gene has been detected in grass carp, milkfish, and tilapia [35,39,40]. In addition, the *scd* and *scdb* genes have been identified in zebrafish, and they have been found to have high homology in humans, mice, rats, and zebrafish [29,41]. In the present study, the coding regions of the *scd1* gene were first identified in mandarin fish. Our results show that the complete ORF sequence of the *Sc-scd1* gene was 1002 bp long and may be used to encode a putative protein with 333 amino acid residues (Figure 5). Sc-SCD1 contained three typical features, namely, four transmembrane domains, two ferrous ion binding sites, and three histidine motifs (aa: 94–100, 131–135, and 272–276), which is similar to the SCD protein in other mammalian species [42,43]. These three histidine motifs are highly conserved among vertebrates [44]. Meanwhile, it has been reported that free ferrous ions maintain the activity of SCD1 in mammals, and they are involved in the regulation of lipid metabolism in organisms [45]. Moreover, multiple protein sequence alignment showed that *Sc*-*scd1* shared a higher homology with *scd1* in teleosts than with that in other vertebrates and that it shared more than 85% identity with other Perciformes [33,44]. These findings indicate that *Sc-scd1* may not only play similar physiological roles but also exhibit functional differentiation in vertebrates.

We conducted a phylogenetic analysis to determine the evolutionary relationships of *scd1* among vertebrates. The results indicate that the *scd1* gene could be divided into two groups: tetrapod and teleost groups. The tetrapod group included mammals, avians, reptiles, and amphibians, whereas the teleost group was further clustered into two adjacent clades, which are similar to a previous study in Nile tilapia [34]. In addition, *Sc*-SCD1 was clustered into one clade with a close relationship with *scd1* in largemouth bass. These results demonstrate that *scd1* was conserved among Perciformes, suggesting that the *scd1* gene may play similar physiological roles in teleosts.

## 5. Conclusions

In the present study, we cloned and characterized the coding regions of the *scd1* gene in mandarin fish and then investigated its expression patterns in response to different nutritional statuses and sexes. Multiple protein sequence alignment and evolutionary relationship, gene structure, and bioinformatic analyses suggested that the *scd1* gene is not only highly conserved but also variable among vertebrates. Additionally, we observed a ubiquitous expression of the *Sc-scd1* gene in all examined tissues, with the liver displaying the highest transcription levels, indicating its potential involvement in the lipid metabolic processes in and feeding habit transformation of mandarin fish. Notably, the distinct tissue distribution patterns of *scd1* under different feeding conditions highlight the influence of varying dietary nutritional profiles on its expression. Our results suggest a potential novel role of the *scd1* gene in the specific domestication of mandarin fish.

## Figures and Tables

**Table 1:** Primer pairs for *Sc-scd1* gene cloning and qPCR.

Primers	Sequences (5′–3′)
*scd1*-01-F	F: GGTTAGGCAGACCATCTTCATC
*scd1*-01-R	R: TCTGGGTTCCCTCACTCTTCC
*scd1*-02-F	F: TACTCTTCTGCCCGTCTTTG
*scd1*-02-R	R: TTCAACAATCTTAGCCACTCC
*scd1*-qF	F: TTGAGAAAGGACGCAAGCTG
*scd1*-qR	R: AAGAAGCACATGAGCAGCAC
*β-actin*-qF	F: AATCGTGCGTGACATCAAGG
*β-actin*-qF	R: TTGCCAATGGTGATGACCTG

**Table 2:** Bioinformation analysis software.

Applications	Softwares	Websites
sequences download	NCBI	https://www.ncbi.nlm.nih.gov/ (accessed on 11 September 2024)
open reading frame (ORF)	ORF Finder	https://www.ncbi.nlm.nih.gov/orffinder (accessed on 11 September 2024)
physicochemical properties of proteins	Expasy	https://web.expasy.org (accessed on 11 September 2024)
protein domain features	Conserved Domain Database (CDD)	https://www.ncbi.nlm.nih.gov/Structure/cdd/wrpsb.cgi (accessed on 11 September 2024)
N-linked glycosylation sites	NetNGlyc-1.0	https://services.healthtech.dtu.dk/services/NetNGlyc-1.0/ (accessed on 11 September 2024)
phosphorylation sites	NetPhos-3.1	https://services.healthtech.dtu.dk/services/NetPhos-3.1/ (accessed on 11 September 2024)
Putative signal peptide predictions	SignalP-6.0 Server)	https://services.healthtech.dtu.dk/services/SignalP-6.0/ (accessed on 11 September 2024)
Putative transmembrane regions	TMHMM	https://services.healthtech.dtu.dk/services/TMHMM-2.0/ (accessed on 11 September 2024)
protein secondary structures	SOPMA	https://npsa.lyon.inserm.fr/ (accessed on 11 September 2024)
protein tertiary structures	Swiss-Model	https://swissmodel.expasy.org/ (accessed on 14 September 2024)

**Table 3:** Composition of the amino acids of SCD1 in *S. chuatsi*.

Amino Acids	Number	Percentage	Amino Acids	Number	Percentage
Ala (A)	29	8.7	Leu (L)	35	10.5
Arg (R)	20	6.0	Lys (K)	19	5.7
Asn (N)	14	4.2	Met (M)	11	3.3
Asp (D)	14	4.2	Phe (F)	23	6.9
Cys (C)	5	1.5	Pro (P)	14	4.2
Gln (Q)	5	1.5	Ser (S)	20	6.0
Glu (E)	16	4.8	Thr (T)	14	4.2
Gly (G)	18	5.4	Trp (W)	10	3.0
His (H)	17	5.1	Tyr (Y)	13	3.9
Ile (I)	10	3.0	Val (V)	26	7.8

**Table 4:** Subcellular localization prediction of SCD1 protein in *S. chuatsi*.

Subcellular Localization	Score	Probability
Cytoplasmic	21.5	48.31
Cytoplasmic/nuclear	12.5	28.09
Plasma membrane	3	6.74
Nuclear	2.5	5.62
Peroxisome	2	4.49
Mitochondrial	1	2.25
Endoplasmic reticulum	1	2.25
Golgi apparatus	1	2.25

## Data Availability

The original contributions presented in the study are included in the article; further inquiries can be directed to the corresponding author.
